# Contributors to positive mental health among youth: a qualitative study in British Columbia, Canada

**DOI:** 10.1093/heapro/daaf127

**Published:** 2025-07-29

**Authors:** Ava Chaplin, Liza McGuinness, Anne Gadermann, Brenda T Poon, Emily K Jenkins

**Affiliations:** School of Population and Public Health, University of British Columbia, 2206 East Mall, Vancouver, British Columbia, V6T 1Z3, Canada; Wellstream: The Canadian Centre for Innovation in Child and Youth Mental Health and Substance Use, University of British Columbia, T304-2211 Wesbrook Mall, Vancouver, British Columbia, V6B 2B5, Canada; Wellstream: The Canadian Centre for Innovation in Child and Youth Mental Health and Substance Use, University of British Columbia, T304-2211 Wesbrook Mall, Vancouver, British Columbia, V6B 2B5, Canada; School of Nursing, University of British Columbia, T201-2211 Wesbrook Mall, Vancouver, British Columbia, V6B 2B5, Canada; School of Population and Public Health, University of British Columbia, 2206 East Mall, Vancouver, British Columbia, V6T 1Z3, Canada; The Human Early Learning Partnership, University of British Columbia, 440-2206 East Mall, Vancouver, British Columbia, V6T 1Z3, Canada; School of Population and Public Health, University of British Columbia, 2206 East Mall, Vancouver, British Columbia, V6T 1Z3, Canada; The Human Early Learning Partnership, University of British Columbia, 440-2206 East Mall, Vancouver, British Columbia, V6T 1Z3, Canada; Wellstream: The Canadian Centre for Innovation in Child and Youth Mental Health and Substance Use, University of British Columbia, T304-2211 Wesbrook Mall, Vancouver, British Columbia, V6B 2B5, Canada; School of Nursing, University of British Columbia, T201-2211 Wesbrook Mall, Vancouver, British Columbia, V6B 2B5, Canada; The Human Early Learning Partnership, University of British Columbia, 440-2206 East Mall, Vancouver, British Columbia, V6T 1Z3, Canada

**Keywords:** youth, mental health, mental health promotion, protective factors, positive mental health

## Abstract

The mental health of youth aged 15–25 is a growing public health concern worldwide, signalling the need for expanded and innovative responses. Approaches to addressing this issue have prioritized treatment and recovery at the individual level, with limited attention to the broader contexts that shape mental health and illness. This study sought to explore youths’ perspectives on positive mental health and its determinants. Drawing on in-depth, semi-structured interviews with a diverse sample of youth aged 15–25 (*n* = 35) from British Columbia, Canada, data underwent thematic analysis guided by a socio-ecological mental health promotion (MHP) framework. Analysis yielded three interconnected themes: (i) perspectives shaped by broader social discourses, (ii) cultivating belonging, and (iii) creating meaning. Participants articulated varying perspectives regarding factors that shape mental health, some of which were grounded in notions of individual responsibility, while others encompassed a wider range of social and political influences. Participants also identified several key contributors to positive mental health, including peer connections that encourage trust, reciprocity, and growth; supportive familial relationships that provide a foundation for wellbeing; access to safe and affirming community spaces for intellectual or creative expression; and engagement in volunteerism or advocacy to establish a sense of purpose and meaning. These findings offer new insights that can extend current responses to youth mental health, including evidence to inform MHP efforts to build and strengthen positive mental health and wellbeing. Findings have the potential to guide policies and programming to enhance wellbeing among youth across the life course.

Contribution to Health Promotion Using qualitative inquiry, this study provides nuanced insights into youth's beliefs and preferences related to positive mental health and wellbeing, highlighting how these perspectives are shaped by broader social structures and conditions. Findings underscore:

- A discrepancy between cultural narratives emphasizing individual responsibility and youths’ lived experiences of the multi-level factors affecting their mental health and wellbeing.
- The need to incorporate youth expertise in identifying relevant supports and in developing policies and programs that improve access to these resources.
- An urgent imperative for holistic population approaches to addressing youth mental health, integrating promotion, prevention, treatment, and recovery.

## INTRODUCTION

Attending to the mental health of youth, often conceptualized as young people aged 15–25, is a critical challenge worldwide [Patel *et al*. 2007, Vaghri *et al*. 2023, World Health Organization (WHO) 2023]. Globally, 13% of youth live with a diagnosed mental health condition (UUNICEF, 2021), while an even greater proportion experience undiagnosed or sub-clinical mental health challenges. Moreover, studies across various geographic regions have demonstrated significant deterioration in youth mental health following the COVID-19 pandemic, prompting newfound urgency to address unmet needs (Racine *et al*. 2021 , Kauhanen *et al*. 2023, Panchal *et al*. 2023).

To date, responses to youth mental health have disproportionately centred on addressing illness at an individual level (Baum *et al*. 2009, Barry *et al*. 2019, Purtle *et al*. 2020). There has been far less attention or investment in efforts to strengthen positive mental health—which constitutes feelings of wellbeing and the capacity to enjoy and engage in daily life activities—for communities and entire populations. Such an orientation falls within the scope of mental health promotion (MHP), a strengths-based approach that directs attention to enhancing individual-, family-, and community-level protective factors, and reducing barriers to wellbeing (Barry *et al*. 2019). This is accomplished, in part, by attending to mental health within everyday settings such as the home, school, or workplace, and by promoting access to resources and opportunities known to improve quality of life and wellbeing (CAMH 2014, Barry *et al*. 2019). MHP aims to establish supportive networks and environments for all, including those already experiencing mental health challenges (CAMH 2014, Barry *et al*. 2019). Researchers and mental health advocates have long asserted that MHP, alongside prevention, treatment, and recovery, is essential to realizing a comprehensive population approach to mental health (Ordóñez and Collins 2015, Purtle *et al*. 2020, Canadian Public Health Association 2021), yet such an approach has not been widely adopted.

Given the historical emphasis on biomedical models of youth mental health in research and practice, there remains limited understanding of youths’ beliefs and preferences related to positive mental health. Previous work has highlighted the need for a more robust evidence base to strengthen the science and practice of MHP, particularly one that prioritizes and integrates youths’ views (O’Reilly *et al*. 2018). Indeed, in recent years, there has been a growing body of research exploring youth perspectives on mental health, including conceptualizations (e.g. Hassler *et al*. 2024), how it should be addressed (e.g. Gard *et al*. 2025), and gendered experiences (e.g. Burke *et al.* 2022). This research is aligned on the complex and socially embedded nature of mental health, and utilizes a variety of frameworks and theoretical perspectives, such as intersectionality and social capital, to unpack and make meaning of youths’ insights. Few studies, however, have sought to elicit youths’ views on the role of broader social, environmental, economic, and political systems in shaping their mental health and wellbeing. This research is essential to guiding efforts that target the social determinants of youth mental health, facilitate access to key resources without compounding existing disparities (Gard *et al*. 2025), and address relevant barriers, ultimately promoting equity. Such evidence has the potential to inform the development of policies and programs that comprehensively respond to the mental health and wellbeing needs of youth. The objective of this qualitative study is to contribute to bridging this research gap.

## MATERIALS AND METHODS

### Theoretical perspectives

This study is guided by a socio-ecological perspective on health and wellbeing. Socio- ecological theories situate individual health-related behaviors and outcomes within broader structures and social systems that contribute to risk or protection (Bronfenbrenner, 1977, McLeroy *et al*. 1988, Stokols 1996). This prompts the exploration of a range of social, political, and environmental factors underpinning health and health inequities (Eriksson *et al*. 2018).

**Figure 1.**
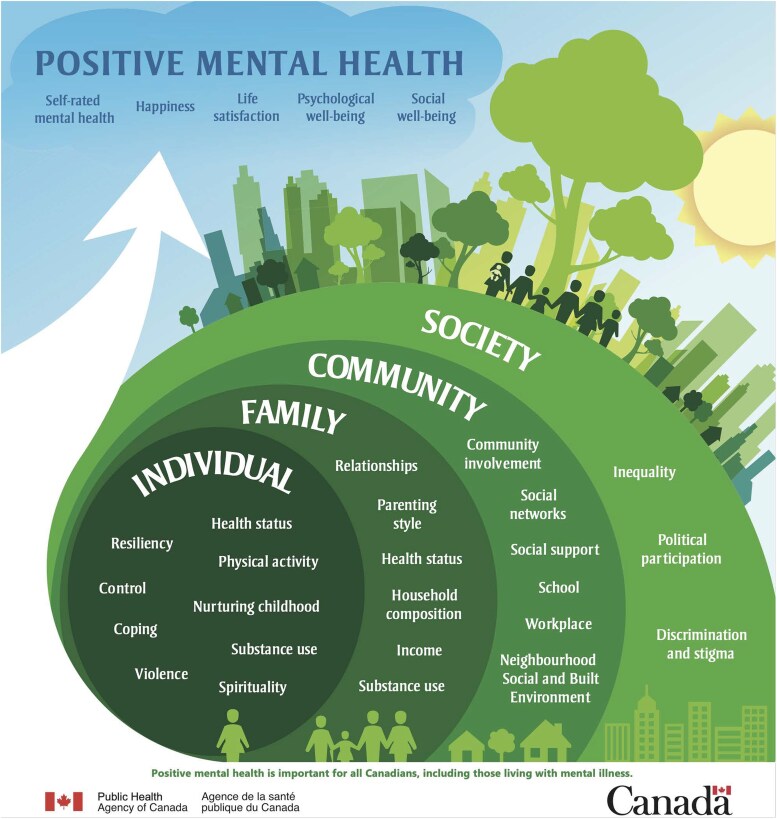
Positive mental health surveillance indicator framework. Orpana *et al*. (2016).

This study draws specifically on the Public Health Agency of Canada's conceptual framework for positive mental health surveillance (Orpana *et al*. 2016), which includes four socio-ecological levels spanning the individual, family, community, and society (Fig. 1), and directs attention to the ways that youth mental health is shaped and can be supported within everyday life settings.

### Study context

This study utilizes data collected as part of an ongoing MHP research and intervention programme, Agenda Gap (Jenkins *et al*. 2020, 2023). Developed in partnership with a diverse group of youth, Agenda Gap seeks to enhance young peoples’ capacity to lead policy change efforts to strengthen the conditions that support mental health for youth and their communities. Before joining the programme, youth aged 15–25, are invited to participate in an interview to explore their social support networks, community contexts, and understandings of mental health. This study used the interview data collected in 2019 and 2020 from youth participating in the Agenda Gap programme in the Lower Mainland region of British Columbia (Bc), Canada.

### Data collection

A purposive sampling approach was used to recruit youth encompassing various social positions and identities, including youth who have accessed mental health services, experienced social hardships (e.g. poverty, child welfare involvement), or who are newcomers or immigrants, racialized, Indigenous, and/or LGBTQ2+. Participants were recruited through partner organizations, which included schools, community organizations, and health services settings, utilizing social media posts, email, and on-site advertisements. Eligibility criteria included being between the ages of 15–25, English speaking, and living in the Lower Mainland region of Bc. Ethics approval was granted through the Behavioural Research Ethics Board at the University of British Columbia (H17-001602).

In-depth interviews took place from April 2019 to October 2020 and lasted between 45 and 90 min. Interviews were conducted by two women, both of whom were experienced in working with youth and trained in qualitative interviewing. Early interviews occurred in-person at a location of each participant's choice (*n* = 15), while later interviews moved to online (i.e. Zoom) due to the onset of the COVID-19 pandemic and related public health restrictions (*n* = 20). Prior to commencing the interview, participants reviewed the consent form with the interviewer and were invited to ask questions. They were informed that their responses would be kept confidential and that they could skip interview questions or withdraw from the study at any time. Participants were also provided with the Know Your Rights with Research tool, developed to support youth in understanding their rights in research participation (Chabot *et al*. 2012). In recognition of youths’ capacity for informed decision-making, youth participants provided their own written or oral informed consent before beginning the interview. All participants received a $20 CAD honorarium. Using a semi-structured guide, interviewers sought to elicit youths’ perspectives on factors that contribute to feeling supported in social and community contexts (see Supplementary File). Participants were also encouraged to share their beliefs and understandings about mental health. Where difficult incidents were shared, participants were encouraged to explore how broader social and structural factors shaped their own or their peers’ experiences, rather than detailing the experiences themselves. Interviewers offered breaks and reviewed options for support on the rare occasion when the interviewer judged it appropriate. All interviews were audio-recorded, transcribed, and anonymized.

### Data analysis

Interview transcripts were uploaded to NVivo12 to support data management. Transcripts and associated field notes were read line by line to develop data familiarity. Thematic analysis was used to support an exploratory, iterative, and reflexive analytical process compatible with addressing the study objective (Braun and Clarke 2019). Through an open-coding process, codes were initially developed describing protective and risk factors identified by participants, and recurrent opinions, beliefs, or ideas about mental health across interviews. As engagement with the data deepened, codes were iteratively grouped and refined to generate initial themes that reflected shared patterns of meaning and that captured connections both within and across socio-ecological levels. Throughout this process, transcripts were frequently revisited to bring further nuance. Initial themes were reviewed through conversation between the authorship team. Final themes and sub-themes were created to reflect how youths’ experiences and perspectives regarding mental health and wellbeing are shaped by factors across socio-ecological levels of influence. In conducting and presenting this analysis, consistent efforts were made to practice reflexivity, including through considering positionality, centring participants’ experiences, immersing in the data and Agenda Gap programme delivery, and generating thick descriptions.

## RESULTS

A total of 35 youth participated in a qualitative interview prior to beginning the Agenda Gap programme (mean age = 17.5 years). Table 1 provides an overview of participant demographics. Several participants had histories of significant trauma, including child welfare system involvement, and many experienced socioeconomic hardship and housing precarity. Findings were constructed and organized thematically into three sections: (i) youth perspectives on mental health as shaped by broader social discourses, (ii) cultivating belonging, and (iii) creating meaning.

**Table 1.** Demographic characteristics of participants.

	Total participants (*n* = 35)
Age in years, *n* (% of total)	
15–18	25 (71.4%)
19–21	3 (8.6%)
22–25	7 (20.0%)
Mean = 17.5	
Gender, *n* (% of total)	
Woman	26 (74.3)
Man	4 (11.4)
Non-binary	3 (8.6)
Undisclosed	2 (5.7)
Ethnocultural identity, *n* (% total)^a^	
White	8 (22.9)
Chinese	7 (20.0)
South Asian	7 (20.0)
Indigenous	5 (14.3)
Black	3 (8.6)
Middle Eastern	3 (8.6)
Vietnamese	3 (8.6)
Other ethnocultural identities	7 (20.0)

^a^Participants’ self-identified and were able to list multiple ethnocultural identities.

### Youth perspectives on mental health as shaped by broader social discourses

Across interviews, participants articulated various perspectives about the ways that mental health and wellbeing are shaped within youths’ lives. These views were informed by wider cultural discourses and ranged from ideas foregrounding personal responsibility to those underscoring the influence of social and political contexts. Occasionally, tension arose as some participants attempted to reconcile their beliefs, which held contradictions reflecting discrepancies between their own experiences, what they witnessed in others, and societal messaging about mental health.

Many participants emphasized the role of personal choice and responsibility, highlighting how they perceived youths’ decisions and behaviours as shaping the trajectory of their mental health and wellbeing. Such decisions were described as including: the ‘choice’ to entertain negative thought processes; the ‘choice’ to confide in others; the ‘choice’ to use recreational drugs; and the ‘choice’ to seek professional care, among others. Participants also expressed the belief that there is a limit to how much an individual can benefit from external sources of support, suggesting they must also make the decision to care for their own wellbeing or to ‘get better’. As one participant shared:

I think that the individual needs the right support and guidance to help them address the issues. But we can only do so much. So, after providing the right support, it's their individual responsibility to take care of their health. (Participant 110, 17-year-old woman)

Another participant articulated that it is difficult to know how someone else is feeling or to predict their mental health needs. She suggested that individual youth need to take responsibility for advocating and caring for themselves:

I'm not going to look after your mental health. I'm not going to be the one being like you have shit mental health. ‘Cause I don't know what that looks like for you. You have to be the one to really look after it … You have to look after it for you. (Participant 131, 25-year-old woman)

Participants endorsing notions of personal responsibility framed their peers as ‘experts of themselves’, in charge of solving their own mental health challenges. Accordingly, these participants tended to champion traits such as resilience, moral fortitude, and empowerment. For instance, one participant shared the following reflections about maintaining a sense of happiness:

I try to take more care of myself … Though it's awesome to have friends, the only way you can maintain friendships is if you can first maintain yourself or if you can first manage how you feel and make yourself happy before other people can. (Participant 111, 17-year-old man)

These beliefs stress the importance of ‘self’ in creating or achieving wellbeing, as in ‘self-care’, ‘self-expertise’ and ‘self-control’. However, by adhering to such conceptualizations, some participants inadvertently attributed blame to individuals who are ‘unsuccessful’ in finding solutions to their challenges, or in other words, those experiencing poor mental health. Moreover, this framing isolated individuals’ experiences from their wider life circumstances, instead positioning them alone in navigating health, illness, and recovery.

Other participants, some of whom simultaneously endorsed notions of individual responsibility, conceptualized mental health challenges as stemming from broader social conditions, including experiences of trauma, a lack of strong interpersonal relationships, feeling unwelcome in their community, and inadequate support systems. This perspective was particularly evident among those who had been in the care of the child welfare system, or who had experienced socioeconomic hardship or housing precarity. Those with these lived experiences recognized the influence of biological factors but suggested that a person's mental health is also strongly influenced by their community context. As one participant explained:

I think the personal side is that you can have a personal disposition, and then if you're put in the right community, you might have good mental health just because you have a great community. Or, if you possibly have a different community, your mental health might not be as great because of that personal disposition. (Participant 132, 16-year-old woman)

Many participants who espoused a more contextually embedded understanding of mental health also emphasized that a strong support network is required to feel motivated to access support and to overcome the substantial psychological and practical barriers associated with seeking professional care for mental health challenges. This sentiment was captured by a participant who shared:

There's this idea that you have to want to get better, which is true, but a lot of people just kind of say ‘well, it doesn't seem like you're trying’ … ‘It seems like you just want to stay the same’. Which, who wants to be in crisis? I think that it takes the community or other people to bring someone to that point where they can want it and they can start accessing the resources they need. (Participant 143, 20-year-old non-binary youth)

Some participants articulated frustration with cultural narratives that undermine experiences of mental health challenges by over-emphasizing choice and suggesting that those who report poor mental health are seeking attention. As one participant expressed:

… I can guarantee you that there's a lot of us, if we had had the choice … I certainly wouldn't have picked to have depression … It's nothing to do with looking for attention. It's the fact that we've gone through trauma, and because of that trauma, this is the result of what we have to deal with. (Participant 140, 22-year-old non-binary youth)

Another participant described how they encounter difficulty receiving care owing to acting as a self-advocate and presenting as capable and assertive:

I would tell her [the school nurse], maybe I look like I'm presenting well right now … but most days of the week I'm at home in bed. The only reason I'm presenting well is ‘cause I'm here… When you are advocating for yourself they say, ‘Well you seem fine to me’. ‘These resources are for people who seem less functional than you look right now’. But when you aren't assertive you don't get anywhere either. It's definitely a catch-22. (Participant 14320-year-old non-binary youth)

Thus, in this example, displaying characteristics typically praised by those who subscribe to narratives of individual responsibility hindered the participant from accessing essential care, creating a situation in which success seemed unattainable and prompting feelings of powerlessness. During some interviews, it was recognized that social inequities, such as a lack of access to basic human needs such as housing, food, and water, can substantially influence mental health. To address such inequities, participants called for community-based approaches and government-led responses, which they indicated are currently inadequate. One participant emphasized the need for systemic change, alongside community- and individual-led efforts:

It's definitely a community response … Especially at the government level as well, not just the individual level, because there are so many individuals here who really do care and make a difference, but that's not enough to actually create systematic change [sic]. We need a government-level change, which isn't happening really. (Participant 109, 17-year-old woman)

Participants’ beliefs about mental health highlight a tension between narratives that emphasize individualistic conceptualizations and those that recognize broader structural and community-level influences, grounded in connection. In some instances, participants acknowledged negative consequences stemming from endorsing individual responsibility for mental health and called for holistic, population-level approaches.

### Cultivating belonging

As participants elaborated on their social and community contexts, nearly all spoke of their peers as consistent and reliable sources of support, vital to promoting and maintaining their mental health. As such, many participants identified their friends as their first point of contact when faced with a mental health challenge, stating that they would feel too afraid or embarrassed to turn elsewhere.

Participants particularly appreciated that their friends tended to share similar values and experiences, whether in regard to schoolwork, hobbies, religious beliefs, sexual orientation, gender identity, or navigating mental health needs. They explained that sharing these characteristics led to an unparalleled level of trust and understanding, particularly when compared with interacting with some adults, including mental health service providers. A participant illustrated this by stating:

Teenagers don't really necessarily look at adults and go, ‘oh yeah, you're someone I can rely on here’. ‘Let me trust you with my issues’. That doesn't really happen … But we do trust each other … We feel as though we are understood by each other because we're the same age and maybe we have gone through similar things. (Participant 122, 15-year-old woman)

Participants conveyed that sharing this trust with their friends allowed them to be vulnerable and candid outside of a professional care setting. Their familiarity with one another also allowed for sensitivity towards each other's boundaries and emotional triggers, fostering meaningful and respectful discussions. It was also noted that, in addition to in-person interactions, social media offered a valuable communication channel for these conversations.

Many participants emphasized that they enjoyed the mutual and reciprocal nature of their friendships, including supporting each other through tough times, inspiring resilience, compassion, and leadership, exchanging outlooks, and sharing resources. One participant provided the following reflections on friendships and their role in wellbeing:

My friends, they don't always have the best advice, but they know what they're doing in terms of offering that community and openness … . I like that space to be vulnerable. How mutual it is and how unconditional it is … (Participant 128, 15-year-old woman)

Additionally, participants shared that their friends provide them with protection from loneliness and bullying, encourage positive self-esteem, and bring joy through fun experiences. Thus, peer connections facilitated opportunities for shared understanding and mutual support, in turn augmenting participants’ sense of self and their ability to cope with daily challenges and enjoy life.

Participants also recognized the importance of support within their family contexts for fostering positive mental health. Many described their relationships with their parents as instrumental in building and sustaining their wellbeing, with some characterizing their parents as the most important figures in their lives. Family support that enabled the youth to have their fundamental needs met contributed to a sense of security that spanned across various aspects of their lives and provided a strong scaffolding for their mental health. Participants also identified instances where their parents advocated for them, helped them navigate the mental health system, introduced them to different coping strategies, and embraced opportunities for learning and self-reflection to best support them. One participant shared that by demonstrating a commitment to introspection and an openness to her interests and path, her parents cultivated a supportive and understanding environment, enabling her to explore her identity confidently:

My mom and dad have been my number one support system … Even though I'm taking a completely different path than what they're doing, they're so open-minded about it, and so supportive. They're researching themselves, learning something new and they're by my side, even though it's different from what they experienced … Since we're all learning together, we're all growing together, it's really strengthened our relationship. (Participant 126, 17-year-old woman)

While family relationships were often foundational and protective for mental health, they also had the potential to contribute to poor mental health. Some participants described strained relationships with immediate family members, which reportedly stemmed from difficulty understanding, agreeing with, or relating to one another. Generational or cultural differences were identified as contributing to gaps in understanding regarding youth's role in family and community, appropriate forms of emotional expression, and mental health. At times, these differences manifested as a lack of sensitivity from parents to the challenges their children encountered, characterized by a ‘tough-love’ attitude, reluctance or inability to discuss emotional matters, and a strict and inflexible approach to discipline. Understandably, strained familial relationships had a particularly profound negative impact on participants’ mental health when they led to frequent arguments, instances of emotional or physical abuse, or an absence of support.

Throughout our interviews, daily life conditions that contributed to stress, including competitive and demanding academic environments, difficulties in balancing various responsibilities, and multiple and competing expectations from parents, teachers, and other adult figures, were regularly highlighted. To seek relief from daily life stressors, participants described engaging in various athletic and artistic pursuits. These activities were reported to provide structure, healthy and productive distractions, and opportunities to meet or spend time with friends, enabling participants to establish a sense of positive functioning and connectedness. Some also referred to listening to music, playing an instrument, or singing as cathartic experiences that helped them process difficult emotions, such as grief, pointing to the potential for art to serve as a powerful self-care tool.

Participants also spoke of the importance of enjoyable community spaces, such as libraries, places of worship, cafes, drop-in centres, and recreational facilities. Many described feeling particularly safe and at ease in community spaces that celebrate diversity and encourage self-expression and authenticity. One participant reflected on the importance of their faith and places of worship in facilitating their mental health:

As a Muslim, I'm really devoted to my religion. And I feel like the mosque is a great place to connect with the community, meet other youth like me, and just, you know, be closer to my religion … It feels really welcoming and it feels good to be accepted and free to follow the religion you want to. (Participant 125, 16-year-old woman)

Participants valued multi-generational environments where their contributions were acknowledged and taken seriously. Such recognition enabled them to better realize their own skills and abilities, and to engage more fully in their surroundings. For example, one participant described her joy upon receiving an award recognizing her contributions to her school community:

I won a little Aboriginal student award and it was nice because I always ate lunch in the Aboriginal Support Room … I was there every day hanging out with them, doing stuff, and it was nice to be valued, like you actually did something, you really contributed to the community here … There were just really supportive adults there and it was a nice, cross-generational kind of thing … It was just a really supportive community in general … When I did get the award, it was like, wow, you guys noticed me. (Participant 109, 17-year-old woman)

Additionally, participants frequently spoke about the beauty and sense of peace offered by natural environments, such as parks, trails, and beaches. Engaging with nature was an essential part of many participants’ routines and contributed to promoting and restoring wellbeing. To this point, one participant reflected on the role that outdoor activity plays in their mental health:

I also have the bike trail which is a very significant place as this is somewhere I go to relieve the stress of school and clear my mind at the end of the day. It's a place where I feel at ease mentally, I can go to clear my head, as well as burn off some energy. (Participant 118, 16-year-old woman)

Throughout the interviews, participants expressed a strong desire for well-connected and tight-knit communities. The need for more spaces, both formal and informal, to connect with peers was repeatedly underscored. This included more community organizations, school clubs, arts programmes, and educational workshops offering programming aligned with youths’ interests, alongside spaces to simply gather and lounge. Several participants also stated that they would like to better know their neighbours and have more opportunities to meet other families and elders in their communities. One participant described the inherent sense of comfort derived from being recognized and greeted by fellow community members. Another articulated:

I feel like as a society we stopped becoming a community and we've segregated ourselves into our own little silos of loneliness … But not that long ago, man, you were able to just go anywhere and talk to your neighbour and feel comfortable with it … I don't even know if we should even be using [the word ‘neighbour’] anymore because, they're just someone you live next to, someone you don't know; they're not a neighbour. We're not a community. When you do find those communities though, you want to hold onto them so tight and squeeze them to death because you're like, this is a place in this world where there's actually people that care about their community and about the things that are happening within it. (Participant 144, 25-year-old woman)

Across interviews, participants highlighted the crucial role of community and belonging in enhancing capacity and promoting mental, emotional, physical, and spiritual wellbeing. They emphasized the value of friendships, close familial relationships, and access to communal spaces for forming ties. Many noted a loss of community connectedness and emphasized the need to foster strong interpersonal bonds and social networks to support wellness.

### Creating meaning—speaking up and giving back

During interviews, a number of participants demonstrated a keen awareness of how inequities tied to race, gender, sexuality, and class shaped their individual lives, as well as their broader communities. A few participants described upsetting or traumatic incidents of discrimination they had experienced, and the emotional and mental health repercussions of these events. Many of these experiences were accompanied by what participants perceived as inadequate institutional-level responses (e.g. from school administrators, employers, or healthcare professionals), which led to further trauma.

Participants also frequently reported that education on mental health and social inequities was greatly lacking in schools, and they indicated that this contributed to ignorance, closed-mindedness, and increased mental health stigma. Consequently, they sought to fill this gap, including by discussing equity with friends, founding school mental health clubs, or taking initiative in educating school staff and other students. Participants described mixed reactions to their school-based advocacy efforts, with some encountering rigidness from community members or an unwillingness to ‘see past their privilege’ (Participant 112, 15-year-old girl). Others described institutional policies on ‘fairness’ that stalled their advocacy efforts. For example, one participant recalled an instance where her peers were initially denied permission to start a ‘Women in Leadership’ club, having been told that it was ‘sexist’ (Participant 128, 15-year-old girl). Those who successfully launched school advocacy initiatives described a sense of heightened empowerment and agency. For example, one participant who contributed to forming a mental health club at her school reflected:

With this club, I’ve been able to learn a lot about mental health … I feel with this group, I'll be able to put my voice out and we can actually make a change … I’m so excited. (Participant 126, 17-year-old woman)

Several participants also shared that they deeply enjoyed ‘giving back’ through volunteer work in their community, which had a profound impact on their mental health. Indeed, participants emphasized that helping others through volunteerism provided them with purpose and meaning, and enabled them to meet new people, access new opportunities, and feel happier, more confident, and more productive. One participant summarized that volunteering allowed her to ‘feel good from the inside’ (Participant_112, 15-year-old girl), a sentiment echoed across many interviews. Another described:

Every summer we have the soup kitchen and it's just really refreshing to be able to cook, talk with friends, and know that you're doing it for a good cause … It just helps alleviate a lot of the trouble I might be having in the background … It's just really volunteering to a) feel good about giving back to the community you've benefited so much from and b) know that you can at least distract yourself productively … Those volunteer opportunities have helped me a lot in the past while. (Participant 111, 17-year-old man)

Participants expressed enthusiasm for participating in community-based activism, viewing their involvement as a way to act against injustices and counter feelings of hopelessness and despair. Several highlighted their engagement in leadership programmes or in youth advocacy groups. This included participants who had been in the care of the child welfare system, or who had experienced homelessness and were now working on related youth advocacy efforts. Participants explained that engaging in such initiatives instilled a sense that they were effecting positive change, making them feel valued. They described being particularly inspired by hearing the stories and ideas of their peers, as this allowed them to feel less isolated in their experiences of hardship. One participant shared their experience as follows:

When we go with [name of organization] to events that we do and the other things I've been part of where you get to hear the stories … It helps all of us, because then we know that it's not just us and that there's more people in our general community who have gone through something similar … It's not just us being treated horribly, it's this person and this person … It grows a bigger community. (Participant 140, 22-year-old non-binary youth)

Similarly, some spoke of the validation they have received when sharing their stories, or ‘using their voice’. Participants indicated that having forums to share their experiences allowed them to explore and further develop their sense of self. One participant emphasized that youth voices deserve to be heard by those in positions of power, and that they are needed to construct healthy communities:

Youth are such a big part of the community. Youth are much more powerful than they were before. I think this generation is really doing amazing things … We can offer so much to the government and the government can gain so much from listening to what we need. (Participant 128, 15-year-old woman)

Across our findings, participants indicated that actions directed towards the welfare of others could simultaneously promote personal wellbeing. This effect appeared particularly pronounced among youth facing social hardships. For them, civic engagement offered a way to challenge systems of oppression and provided valuable opportunities to share their lived experiences and collaborate with or obtain support from peers.

## DISCUSSION

Youth mental health is a growing public health concern that calls for new and innovative strategies (Vaghri *et al*. 2023). This study adds to a body of literature that acknowledges the need for youth expertise in addressing this issue. Indeed, the WHO has long asserted that youth's perspectives should be incorporated in the development of policies and programmes that affect them (WHO 1999). Previous research has identified a range of individual, family, community, and societal conditions with the potential to positively influence mental health and wellbeing (Orpana *et al*. 2016). This study offers rich insights into how youth conceptualize mental health, with a focus on positive mental health and wellbeing, including context-sensitive considerations and the role socio-structural forces, which hold important implications for advancing the MHP field.

Findings from this study indicate that youth often struggle to reconcile tensions between cultural narratives that position health as an individual responsibility and perspectives shaped more directly by their lived experiences. This includes experiences of connecting with friends and family, accessing safe and inclusive spaces in community, and engaging in civic causes. Indeed, the narratives of individual responsibility surfaced in this study can be linked to broader social norms tied to neoliberal ideologies, which have been prominent in Western society since the 1970s (Crawford 1980, Greco 1993, Gill and Orgad 2018). According to neoliberal thought, health is a choice that lies within an individual's control. As free and rational agents, individuals are not only expected to pursue health, but bear a civic and moral obligation to do so (Crawford 1980). Within this paradigm, ‘self-care’ equates to a set of behavioural expectations demanding adherence and vigilance (Gill and Orgad 2018).

Many scholars and mental health advocates have warned of the negative repercussions of this framing on population wellbeing (Crawford 1980, Greco 1993, Gill and Orgad 2018). It has been noted that an emphasis on individual responsibility and capacity for resilience renders health apolitical, effectively suppressing calls for government or other institutions to provide supports. It may also evoke feelings of inadequacy and shame, as it suggests that those experiencing poor health have failed in some manner. This has been well described in HIV/AIDS literature, in which neoliberal approaches to public health intervention (e.g. targeting individual-level behaviours, such as condom usage or sharing of injection equipment) have been critiqued for perpetuating stigma and feelings of isolation among affected populations (Dodds 2002, Krüsi *et al*. 2017 ). Relatedly, a qualitative study examining queer youths’ pandemic experiences described an intense struggle to balance adherence to public health guidelines against a longing for connection (Stehr *et al*. 2024). Participants attributed morality to compliance, and experienced shame for making ‘wrong’ or ‘risky’ choices, highlighting the repercussions of transferring responsibility for managing risk onto individuals. The present study extends these understandings with evidence that upholding individual responsibility ideals for mental health may contribute to greater stigma, stereotyping, and social isolation. This includes perpetuating the idea that those reporting mental health challenges are merely attention-seeking, or that if someone is capable of self-advocating, they are not ‘sick enough’ to qualify for care.

Within this wider social and political context, socio-ecological orientation of MHP can serve as a valuable tool for shifting perspectives from individual-level experiences and illness-based conceptualizations to also centre the influences of broader social and structural inequities (Barry *et al*. 2019). In the realm of education, much public and professional attention has focused on ‘mental health literacy’ (MHL), which involves enhancing knowledge about mental health conditions, their causes, symptoms, and methods of seeking professional care (Kutcher *et al*. 2016). While MHL is an important and valuable approach, the findings of the present study suggest that it may not currently extend far enough in its scope. Indeed, our findings offer support for ‘MHP literacy’, or efforts to improve understandings of the social and structural determinants of mental health (Jenkins *et al*. 2023).

Another key finding relates to the critical role of peers in supporting mental health experiences and outcomes among youth. Youth in this study consistently described the joy, and rewarding feelings of connectedness and belonging cultivated through peer relationships. Moreover, youth participants demonstrated that they bring unique strengths and expertise to supporting one another, building community, and promoting inclusive environments. This evidence suggests that programming that includes opportunities for peer connection, learning, and sharing is highly valuable towards promoting positive mental health at this developmental stage. Recognizing the value of peer relationships and the importance of reciprocity and trust has prompted the uptake of youth peer support models (Simmons *et al*. 2023, Murphy *et al*. 2024). The present study supports further investigation into both formal and informal peer-support opportunities for youth. This includes efforts to better understand how peer support can be strengthened within the settings that youth occupy on a regular basis, and how such environments can better monitor and address disruptions in peer relationships and friendship groups that may impact feelings of connectedness and belonging (Gowing 2019, Halsall *et al*. 2022). Paired with evidence of youths’ interest in advocacy, our findings suggest that peer support models that incorporate a social justice and systems change focus might be particularly well suited to this age demographic.

The study findings further highlight the potential for civic engagement, particularly in the form of volunteering and activism, to enhance young peoples’ social, emotional, and mental wellbeing. A substantial body of literature has explored the influence of civic engagement on various mental health outcomes (Flores *et al*. 2018, Fenn *et al*. 2024). Among older adults and adolescents, evidence indicates positive associations between civic engagement and mental health, although identifying causal pathways remains a challenge (Fenn *et al*. 2024). Relatively little research has investigated this phenomenon among youth, particularly those from groups that have endured systemic and structural hardship (Ortega-Williams *et al*. 2020, Fenn *et al*. 2024, Gard *et al*. 2025). The present study contributes to this literature by featuring the perspectives of diverse youth, including those who have experienced significant adversity, and where the relationship between efforts to challenge oppression and improved wellbeing appears particularly prominent. Further research in this area is needed; however, as there is evidence of potential drawbacks to civic engagement, particularly related to the investment of time and energy required (Flores *et al*. 2018, Fenn *et al*. 2024). This includes the need for additional research exploring civic engagement within MHP interventions, particularly with a focus on more well-established interventions (e.g. those that extend beyond initial pilot phases), and on uncovering long-term feasibility and programme impacts (Barry et al. 2013, Jenkins *et al*. 2023).

Research indicates that across communities and regions, approaches to youth mental health remain highly individualistic and biomedical in nature (Hobbs *et al*. 2024). This was true in the communities from which youth participants were recruited for the present study. Investment in efforts to extend current mental health programming beyond this paradigm, to also include initiatives aimed at strengthening the social fabric of our communities and building opportunities for social connection and meaning, could help to more fully respond to youths’ mental health needs (Birrell *et al*. 2025). Such an approach would reflect the diverse ways that youth understand mental health and wellbeing, while centring developmental considerations and assets (Humphrey and Bliuc 2021). This study has several strengths and limitations, capturing youth perspectives on factors contributing to mental health across varied youth and community settings. Nevertheless, the potential for sampling and social desirability bias should be acknowledged. For example, participants consisted of youth who opted to participate in the Agenda Gap intervention, and thus, may have had a pre-existing interest in mental health advocacy and a propensity for civic engagement. Furthermore, given their interest in participating in the Agenda Gap programme, participants may have felt inclined to discuss their perspectives on mental health in ways that they believed would most align with the study's objectives. However, the range of perspectives shared, including conflicting beliefs among and across participants, suggests that this issue had minimal impact on the study findings. Additionally, the sample was comprised of youth living in urban and suburban communities and included a disproportionate number of participants who identified as girls/women. Further research is needed to understand how youth's perceptions and beliefs related to positive mental health differ according to non-programme and geographic settings (i.e. within urban, suburban, and rural geographies) and across gender identities.

## CONCLUSION

This study illustrates that youth experience a disconnect between societal messages emphasizing individual responsibility for mental health and their lived experiences of the multi-level factors that shape and influence wellbeing. The findings of this study have several implications, particularly in relation to how we might reconsider mental health messaging and education, and how we can more effectively foster supportive community environments and social networks for youth, including by better integrating and building upon their existing resources for positive mental health. Findings suggest that responses exclusively focusing on biomedical interventions are not sufficient for addressing youth mental health. To comprehensively respond to this pressing public health issue, interventions that are grounded in MHP and that aim to strengthen or facilitate access to protective factors are required, alongside prevention, treatment, and recovery efforts.

## Supplementary Material

daaf127_Supplementary_Data

## Data Availability

The data underlying this article cannot be shared publicly due to its sensitive and potentially identifying nature. The data will be shared on reasonable request to the corresponding author.
