# Supplementary material for: Contributors to positive mental health among youth: a qualitative study in British Columbia, Canada
Source: Health Promot Int. 2025 Jul 29;40(4):daaf127. doi: 10.1093/heapro/daaf127 (PMC12304415; doi:10.1093/heapro/daaf127)
Supplement: daaf127_Supplementary_Data [file daaf127_supplementary_data.doc]

**Interview Guide**

1. **SOCIAL & FAMILY CONTEXT**

**To begin, I’m wondering if you could draw a diagram of the 5 or so most important people in your life? This might be a friend, family member, teacher, or neighbour. Whoever is important to you.**

Probes:

- 1. Can you tell me about the people you’ve listed?
  2. Why are they important to you?
  3. Can you describe what it’s like to live in your community?
  4. What’s good and bad about living here?
  5. Have you always lived in this community?
  6. Who do you live with?

1. **COMMUNITY CONTEXT**

**Like you just did, could you draw me another diagram, but this time include the 5 or so most important parts of your community, the things that make you feel good or safe (either physically or emotionally)? (e.g., parks, schools, community centres)**

- 1. Can you tell me about the places you’ve listed?
  2. Why are they important to you?
  3. Is an online community or friend network important to you?
  4. Are there situations or places that make you feel unsafe, either physically or emotionally, in your community, including online?
  5. What’s life like here in this city and neighbourhood?
  6. Thinking about the community you live in or a mentally healthy community, what would you see, touch, do, hear?
  7. How does who you are - characteristics like your gender, age, ethnicity, disability or diagnosis, or substance use - make a difference in your experiences of, or what you notice, about your community? (What do you notice/experience that others might not?)

1. **COMMUNITY CONNECTIONS**

**Tell me about the services and supports in your community that you use.**

Probes:

- 1. What’s good about these services?
  2. What aspects of your community work well for you?
  3. Can you tell me about where can you go in your community if you need support? What has supported you in accessing supports? What has been a barrier?
  4. What would you like to see offered in your community that would support young people?
  5. Can you tell me about a time you took action and it went well?

1. **YOUTH MENTAL HEALTH**

**We know from other research we’ve done that young people are dealing with some difficult experiences. It seems everyone has good days and bad days, too. Can you tell me about a time when you were stressed, worried, or particularly “emotional?” Can you start from the very beginning and tell me as much about the experience as possible?**

Probes:

- 1. What was going on in your head at that time?
  2. When did those feelings start?
  3. How did you cope?
  4. Who did you talk to and how? (online, in-person, with family, friends, teachers, counselors, doctors)
  5. What resources did you need to help you cope in this situation?
  6. What could your community do to help you cope?
  7. What aspects of your community make you feel good?
  8. Are there services here for youth when they have tough days?
  9. What could this community/neighbourhood/city do to better support youth when they have tough days?
  10. Thinking about who you are, what was your experience of this (e.g., as a girl/boy, member of a minority group, youth in the foster care system, etc.)? Do you think other kids have similar experiences?
  11. How, if at all, have existing systems (education, community programs, social and health services, contributed to your experiences?
  12. How was the experience you described shaped by _____? (i.e. your neighbourhood/community, the child welfare system, etc.)

1. **PROMOTING MENTAL HEALTH**

**Can you tell me about a time you felt particularly valued or supported?**

Probes:

- 1. What was going on?
  2. What makes this experience stand out?
  3. Did anything or anyone prompt this experience (if so, who/how?)
  4. What’s one thing that could make your life better right now?

**Policies are a tool that governments use to guide how communities are structured and operate. Can you think of any examples of how government “rules” have influenced your experiences or access to supports?**

a. Have you or your family ever come up against a rule that made life hard or created problems – or solved problems?

b. How did that impact your stress or worry?
